# Genomic characterization and clinical evaluation of prosthetic joint infections caused by *Cutibacterium acnes*

**DOI:** 10.1128/spectrum.00303-24

**Published:** 2024-10-08

**Authors:** C. Liew-Littorin, S. Davidsson, Å. Nilsdotter-Augustinsson, B. Hellmark, H. Brüggemann, B. Söderquist

**Affiliations:** 1 Department of Obstetrics and Gynaecology, Faculty of Medicine and Health, Örebro University, Örebro, Sweden; 2 Department of Urology, Faculty of Medicine and Health, Örebro University, Örebro, Sweden; 3 Division of Inflammation and Infection, Department of Biomedical and Clinical Sciences, Linköping University, Linköping, Östergötland, Sweden; 4 Department of Laboratory Medicine, Clinical Microbiology, Faculty of Medicine and Health, Örebro University, Örebro, Sweden; 5 Department of Biomedicine, Aarhus University, Aarhus, Denmark; 6 School of Medical Sciences, Faculty of Medicine and Health, Örebro University, Örebro, Sweden; Instituto de Investigacion Sanitaria, Madrid, Spain

**Keywords:** prosthetic joint infections, *Cutibacterium acnes*, single locus sequence typing, whole genome sequencing

## Abstract

*Cutibacterium acnes* is a major skin commensal that may act as an opportunistic pathogen. It is difficult to interpret findings of *C. acnes* in tissue cultures obtained during arthroplasty revision surgery, since they may represent true infection or contamination. This study investigated whether *C. acnes* obtained from prosthetic joint infections (PJIs) were related and shared common genomic traits that might correlate with clinical courses and patient outcomes. *C. acnes* isolates from revision surgery of patients with PJIs of the hip, shoulder, and knee were characterized using molecular methods to determine the sequence type (ST) and the presence of possible virulence determinants (Christie–Atkins–Munch-Peterson factors, dermatan sulfate-binding adhesion 1, hyaluronidase lyase, and linear plasmid). A standardized review of the patients’ medical charts was performed. The study included 37 patients with *C. acnes* culture-positive tissue samples where multiple isolates of *C. acnes* belonged to the same ST. Most of the isolates belonged to phylotype IA_1_. Phylogenetic analysis of virulence determinants revealed no shared pattern among PJI isolates. Seven patients had a polymicrobial infection. Exchange revision was performed in 70% of the patients, and >50% of all patients received antibiotic treatment for ≥3 months. Failure was noted in seven patients. No specific ST or any identifiable unique feature among virulence determinants were found among *C. acnes* isolated from PJIs of hips and shoulders. The majority of patients had low inflammatory markers and were treated successfully, even polymicrobial infections. However, failure was more common among shoulder infections compared with hip infections.

**IMPORTANCE:**

Prosthetic joint infection (PJI) is a rare complication after arthroplasty surgery. The infection seldom resolves without a combination of both surgical and antibiotic treatment and can cause significant suffering among affected patients. *Cutibacterium acnes* is a common skin bacterium that is most often found in shoulder PJIs but can also infect other prostheses. In this study, we conducted a review of patients with previously verified PJIs involving *C. acnes* in hip or shoulder prostheses, along with a genomic analysis of the bacteria causing the infections. The majority of patients had successful outcomes. We did not identify any specific phylogenetic lineage or specific molecular signature of virulence factors among these PJI-associated *C. acnes* isolates that seemed to be associated with increased potential to cause infection among this species. This indicates that *C. acnes* isolated from PJIs originates from the patients’ own skin microbiome and is inoculated during the arthroplasty surgery.

## INTRODUCTION

Arthroplasty surgery is a common procedure that provides pain relief and improves function and quality of life. According to the Swedish hip, knee, and shoulder arthroplasty registers, more than 30,000 hip and knee replacements (1) and approximately 2,000 shoulder replacements are performed annually (https://www.ssar-rapport.se/SAAR_web/). However, prosthetic joint infection (PJI) is one serious complication of this surgery. Though rare, PJIs result in increased mortality and substantial morbidity, such as long-lasting pain and permanently impaired joint function (2–4). The risk factors for PJIs are not fully understood but include underlying conditions such as obesity, diabetes mellitus, and rheumatoid arthritis (5–9).

The microbiological diagnostic criteria of a PJI are based on the growth of phenotypically indistinguishable microorganisms in at least two perioperative tissue and/or synovial fluid samples (10). The infection occurs directly after surgery either (early infection) with delayed onset (>3 months to 2 years after implantation) or with late onset (>2 years) (11). Delayed-onset PJIs are still likely to have been caused by pathogens inoculated during the primary surgery, but in this case by low-virulence bacteria rather than more virulent species (12). Treatment options for PJIs consist of both antibiotics and surgical management. Surgical options include a debridement, antibiotics, and implant retention (DAIR) procedure to salvage the prosthesis or a one-step or two-step total exchange revision (13).

*Cutibacterium acnes*, formerly known as *Propionibacterium acnes*, is part of the normal skin microbiota and is commonly found in pilosebaceous follicles of the skin. It has been reported as an opportunistic pathogen, mainly in the pathogenesis of acne vulgaris (14). Several reports indicate that *C. acnes* is an etiological agent of various infections, especially in implant-related infections, including prosthetic valve endocarditis, shunt-related meningitis, and PJIs (15–17). However, *C. acnes* has been demonstrated in native shoulder joints, in samples taken during early stages of the primary joint replacement surgery (18, 19). Thus, a significant issue in evaluating clinical samples is the separation of true *C. acnes* infection from skin-derived contamination.

*C. acnes* can be divided into six main phylotypes: IA_1_, IA_2_, IB, IC, II, and III (20). All types can be found on normal skin, but phylotype IA is enriched on acne vulgaris-affected skin, whereas other types, in particular types IB and II, have been associated both with healthy skin, but also with implant-associated infections (21).

Based on *C. acnes’* core genome phylogeny, a single locus sequence typing (SLST) scheme has been presented, distinguishing 10 main SLST classes: A, B, C, D, E, F, G, H, K, and L (22). SLST classes A–E correspond to phylotype IA_1_, and SLST classes F, G, H, K, and L correspond to phylotypes IA_2_, IC, IB, II, and III, respectively.

Several virulence traits for *C. acnes* PJIs have been described. A *C. acnes* surface glycoprotein known as dermatan sulfate-binding adhesion 1 (DsA1), which recognizes human fibrinogen, has been found among type IA_1_, IA_2_, and IC strains and might contribute to virulence by increasing binding capacity, inflammation, and promoting bacterial survival in the host (23). Furthermore, Christie–Atkins–Munch-Peterson (CAMP) factors 1–5, which are proteins with pore-forming activity, might contribute to hemolysis and increased inflammation (15, 24, 25). Hyaluronate lyase (Hyl) is another putative virulence factor that has been discovered in two separate variants (Hyl-IA and Hyl-IB/II) among *C. acnes* phylotypes (26, 27). It is an enzyme that breaks down glycosaminoglycans and hyaluronic acid, which are key components of extracellular matrix and articular cartridge and hence might facilitate the spread and growth of bacteria in the tissue (28, 29). Moreover, the presence of a linear plasmid has been proposed as a possible virulence determinant of *C. acnes*, since this plasmid harbors a tight adhesion (tad) locus that might enhance biofilm production and enable tight adhesion to abiotic surfaces, thus enhancing colonization (30–32).

In the present study, *C. acnes* isolates obtained during hip, shoulder, and knee arthroplasty revisions were analyzed. Isolates were typed and genome sequenced in order to examine the molecular epidemiology of PJI-associated *C. acnes*. We also investigated whether these strains shared common genomic traits that correlated with the clinical course and patient outcome, and hence that might explain their etiological role in PJIs.

## RESULTS

### Patient and isolate cohort

**Fig 1:**
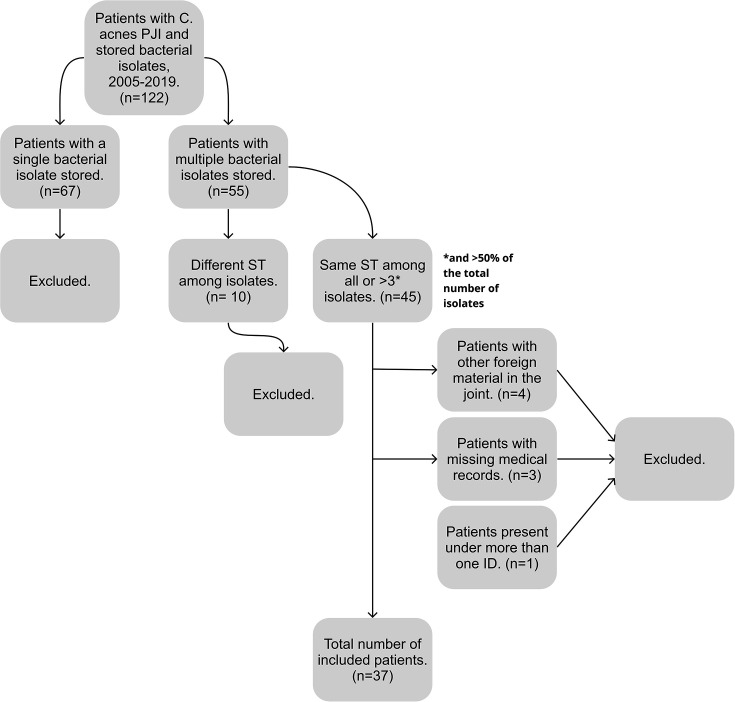
Flow chart showing how patients with PJI caused by *C. acnes* were selected for the present study. *Four patients with identical sequence types (STs) among at least three isolates, which in turn made up >50% of the total number of isolates for each individual patient. See Fig. S1.

In total, 122 *C*. *acnes* tissue culture-positive arthroplasty revisions were initially considered for inclusion. According to the European Bone and Joint Infection Society (EBJIS) definition (10), only patients with ≥2 positive samples with the same microorganism are considered to have a PJI, and thus, all cases with only one *C. acnes*-positive sample (*n* = 67) were excluded, leaving 55 cases in which two or more isolates were stored per case. All isolates (*n* = 150) from these 55 cases were analyzed using SLST. In 45 cases, the same *C. acnes* SLST type was found in at least two of the stored isolates (Fig. 1; Fig. S1); the other 10 cases showed different SLST types in each of their stored strains and hence were excluded (Fig. S2). The 45 remaining patients were intended for clinical evaluation following review of the medical records. However, eight of them had to be excluded, mainly due to inadequate access to medical records or because the review of medical charts revealed that they had other osteosynthesis materials as well as their arthroplasty. The final case group comprised 37 patients for whom at least two *C. acnes* isolates with the same SLST type had been isolated (from multiple tissue samples) and clinical data were available (Fig. 1). Among these, 23 patients had shoulder prostheses, one had a knee prosthesis, and 13 had hip prostheses. In both the hip group and the shoulder group, the mean number of intraoperative tissue samples obtained per patient was seven and the mean number of positive cultures per patient was five. Overall, the mean number of stored isolates per patient was four (range: 2–10). One *C. acnes* isolate from each patient was selected for whole genome sequencing.

For seven (18%) of the included patients, ≥2 of their tissue cultures were positive for other bacterial species than *C. acnes*. Five patients were co-infected with *Staphylococcus epidermidis* (*n* = 3–4 isolates), one patient with both *Staphylococcus saccharolyticus* (*n* = 3) and *Staphylococcus capitis* (*n* = 2), and the remaining patient with *S. saccharolyticus* (*n* = 2) (Table S1). Six of the seven polymicrobial infections were found in patients with shoulder PJIs (*P* = 0.38).

### SLST and whole genome phylogeny of *C. acnes* strains obtained from patients with PJI

**Fig 2:**
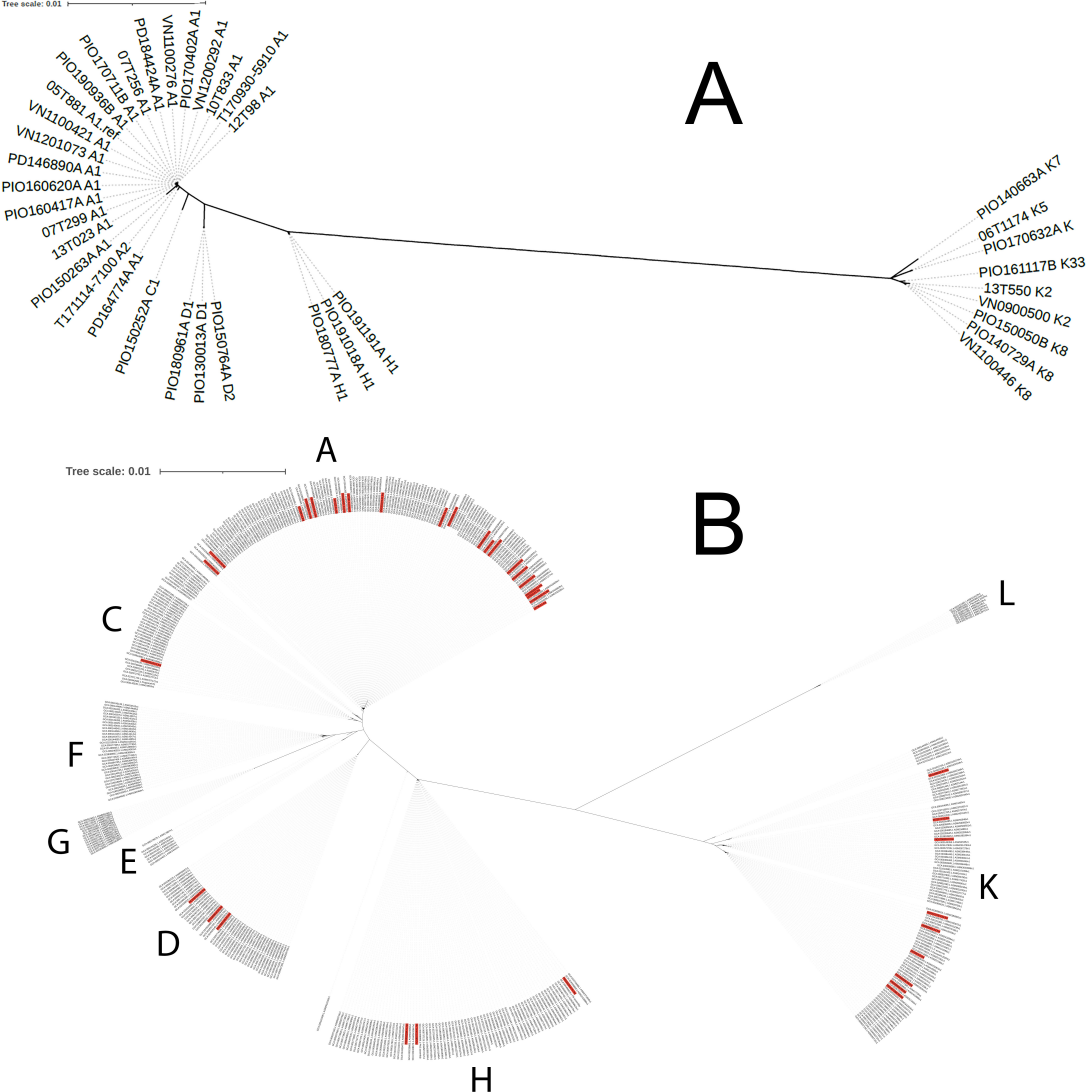
Phylogenetic tree based on core genome alignment of *C. acnes* strains from PJIs included in the present study. (A) The core genome of all 37 strains was compared, and a phylogenetic tree was constructed on the basis of core genome-located single nucleotide variants. The SLST types of the respective strains can be found in the tree (part of the strain name). Most strains were in SLST class A (phylogenetic clade IA1); despite being closely related, they represent distinct strains. See also Fig. S3. (B) The 37 genomes were compared with other publicly available strains (*n* = 438 as of March 2023), including strains from normal skin, acne lesions, and other foreign body infections. The different phylogenetic clades were clearly separated (SLST classes A–L).

The 37 sequenced strains belonged to SLST classes A, C, D, H, and K (Fig. 2A). Of the 21 strains (56.7%) belonging to class A (phylotype IA_1_), 20 were assigned to type A1 and 1 to type A2. The majority of both hip (7/13; 53.8%) and shoulder (14/23; 60.8%) PJI isolates belonged to class A. The nine strains (24.3%) belonging to class K comprised one knee, three hip, and five shoulder PJI isolates. The other strains belonged to classes H (*n* = 3), D (*n* = 3), and C (*n* = 1).

A few clusters of phylogenetically related type A1 strains were identified among the patients, indicating a closer relationship between these strains isolated in Sweden (including strains from both Region Örebro County and Region Östergötland) compared with A1 strains from other geographic regions (Fig. 2B). However, all type A1 strains displayed >100 strain-specific single nucleotide variants, indicating their strain individuality (Fig. S3).

*C. acnes* isolates from polymicrobial infections were also analyzed separately, and no significant association to specific SLST classes could be identified among these isolates. The isolates belonged to SLST classes A (*n* = 4), D (*n* = 1), and K (*n* = 2) (Table S1).

### Putative virulence factors

**Fig 3:**
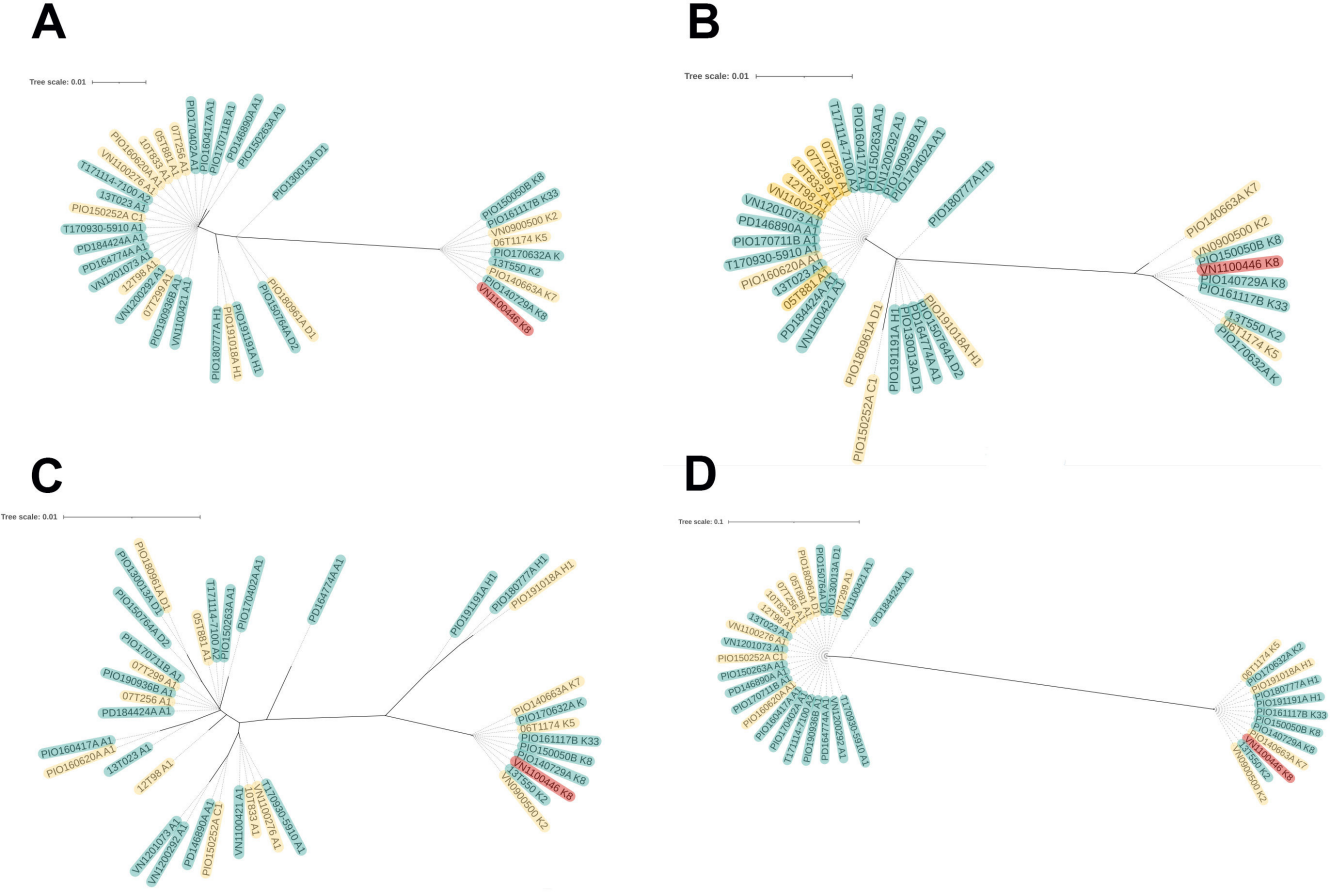
Phylogenetic analysis of virulence factors of *C. acnes* strains isolated from PJIs. The protein sequences of four virulence factors from all investigated isolates were determined for this analysis. (A) CAMP factor 1; (B) CAMP factor 2; (C) surface glycoprotein DsA1; (D) hyaluronidase. Strains from shoulder infections are marked in yellow, strains from hip infections are marked in yellow, and the strain from the knee infection is marked in red in all figures. The ST of each isolate is displayed next to each strain name.

To investigate whether these *C. acnes* PJI isolates shared specific genomic features that differed from other *C. acnes* strains, we analyzed four previously described virulence factors: CAMP factors 1 and 2, DsA1, Hyl, and the linear plasmid containing the tad locus. The phylogenies of CAMP factors 1 and 2 reflected the core genome-based phylogeny (Fig. 3A and B), and we detected no specific sequence differences of these CAMP factors with regard to the origin of the strain (hip or shoulder PJI). The same was found for the phylogeny of DsA1 (Fig. 3C), although the phylogeny was more diversified due to the strain-specific presence of proline–threonine repeats in the DsA1 protein sequence. An analysis of the Hyl proteins revealed two clearly separated clades consisting of phylotype IA (SLST classes A, C, and D) carrying the Hyl-IA variant and phylotype IB/II (SLST classes H and K) containing the Hyl-IB/II variant (Fig. 3D) (26, 27).

**Fig 4:**
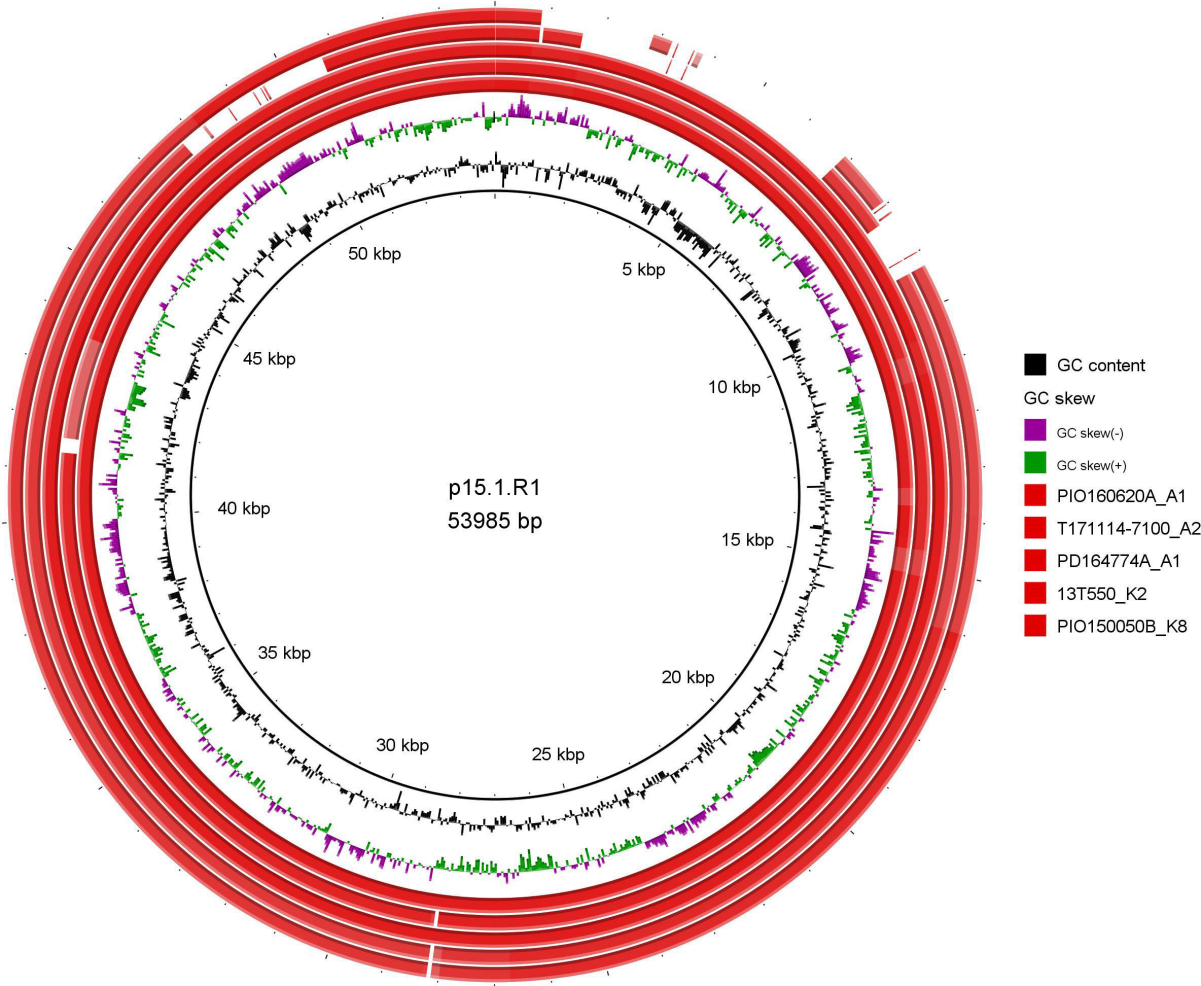
Comparison of five plasmids identified in *C. acnes* isolated from PJIs of the shoulder and the hip. The plasmid p15.1.R1 was used as reference (33). SLST class A strains are represented by the three innermost circles, and class K strains are represented by the two outermost plasmids. Differences between the plasmids from class A and K strains were detected. The class K strains lacked approximately 9 kbp of the plasmid.

Five of the 37 *C*. *acnes* strains carried a plasmid that encoded putative virulence markers, such as the tad locus. Three strains belonged to SLST class A (two A1, one A2) and two strains belonged to class K (K2 and K8) (Fig. 4). The K-type strains lacked approximately 9.25 kbp of the plasmid, which is in line with a previous report by Davidsson et al. (30).

### Patient characteristics

Characteristics of the study population are presented in Table 1. Two-thirds of the patients were male (25/37; 67.5%), and the median age at the time of diagnosis was 66 years (range: 26–81). The majority (24/37; 64.8%) had no significant co-morbidity or risk factor noted in their medical records, and the most common indication for arthroplasty surgery was osteoarthritis (23/37; 56.0%). Fracture as the reason for surgery was more common among patients with shoulder prostheses, but there was no statistically significant difference between patients with hip and shoulder arthroplasties (*P* = 0.21).

**TABLE 1:** Characteristics of patients (*n* = 37) with PJIs in the hip, knee, and shoulder caused by *C. acnes*^*a*^

Patient characteristics	Hip	Knee	Shoulder	Total (%)
Gender				
Male	7	1	17	25 (68%)
Female	6		6	12 (32%)
Total	13	1	23	
Comorbidities and risk factors				
None	9		15	24 (65%)
Smoking			1	
Vascular disease			1	1 (3%)
Alcoholism	1		2	3 (8%)
Malignancy	1			1 (3%)
Diabetes mellitus type 2			1	1 (3%)
Prior joint surgery	2		1	3 (8%)
Anticoagulants			3	3 (8%)
Hypertonia			2	2 (5%)
Asthma	1		2	3 (8%)
Parkinson’s disease		1		1 (3%)
Alzheimer’s disease	1			1 (3%)
Obesity			2	2 (5%)
Indication for primary surgery				
Osteoarthritis	9	1	13	23 (62%)
Rheumatoid arthritis			2	2 (5%)
Fracture/trauma	1		7	8 (22%)
Congenital malformations	1			1 (3%)
Destruction of joint without trauma	2		1	3 (8%)
Type of implant				
Cemented	8	1	9	18 (49%)
Uncemented	4		9	13 (35%)
Unknown	1		5	6 (16%)
Type of surgery prior to infection				
Primary	7	1	19	27 (73%)
Revision	6		4	10 (27%)
Number of surgeries prior to positive cultures (incl. primary), median (range)	4 (2–12)		2 (2–2)	
Polymicrobial infection including *C. acnes*	1		6	7 (19%)

^*a*^ All data are presented as the number of patients, if not otherwise specified.

### Clinical data for patients with PJI caused by *C. acnes*

The most common symptom of PJIs of the hip, knee, and shoulder was pain (31/37; 83.7%), followed by loss of function (12/37; 32.4%), redness (8/37; 21.6%), and swelling (8/37; 21.6%) (Table 2). More acute symptoms such as fever, wound secretion, and abscesses were more common among patients in the shoulder group, who also presented with a larger number of acute infections compared with the hip group (Table 2). We could not identify any statistically significant differences in the presentation of symptoms between patients with monomicrobial vs polymicrobial PJIs (*P* value range from 0.27 to 1). Six patients, one with a hip infection and five with shoulder infections, sought medical care within 30 days postsurgery (range: 3–29 days); however, no patient was diagnosed with a *C. acnes* PJI within 30 days after primary surgery. Two of these patients were later diagnosed with a polymicrobial PJI of the shoulder, one with *S. epidermidis* and the other with *S. saccharolyticus*. There was no statistically significant difference between polymicrobial and monomicrobial shoulder infections regarding classification of acute infections (*P* = 0.65).

**TABLE 2:** Characteristics of PJIs in the hip, knee, and shoulder caused by *C. acnes* in 37 patients^*a*^

Infection characteristics	Hip	Polym. hip	Knee	Shoulder	Polym. shoulder	Total (%)
Classification						
Early onset (<3 months since last surgical intervention)	2			6	3	11 (30%)
Delayed onset (3–24 months)	4		1	6	3	14 (38%)
Late onset (>24 months)	6	1		4		11 (30%)
If >24 months, median time to infection (years, range)	9 (5–15)	15 (15)		4 (4–7)		
Unknown^*b*^				1		1 (3%)
Loosening of implant at radiology?						
Yes	8	1	1	6	3	19 (51%)
No	3			7	2	1 (32%)
No radiology performed	1			4	1	6 (16%)
Issues with wound healing?						
Yes	5			2	3	10 (27%)
No	4	1	1	12	3	21 (57%)
Unknown	3			3		6 (16%)
Symptoms at diagnosis						
Pain	11	1	1	13	5	31 (84%)
Loss of function	4		1	7	1	13 (35%)
Redness	3			2	3	8 (22%)
Swelling	2		1	5	1	9 (24%)
Fever	1			2	1	3 (8%)
Wound secretion				3	2	5 (14%)
Abscess				2	2	4 (11%)
Traumatic periprosthetic fracture	1			1	1	3 (8%)
Number of cultures, median (range)	6 (5–12)	5 (5)	5 (5)	5 (3–10)	5 (5-12)	
Number of *C. acnes*-positive cultures, median (range)	4 (2–9)	4 (4)	5 (5)	5 (2–9)	5 (2-10)	
C-Reactive protein > 5 mg/L, median (range)	73 (26–116)			58 (13–160)	40 (14–113)	
Number of patients with C-reactive protein < 5 mg/mL	6	1	1	8	1	17 (46%)
Not documented	1			3	1	5 (14%)
Blood, leukocyte cell count ≥ 11 × 10^9^ /L, median (range)	13 (13)			11.5 (11–12)		
No. of patients with leukocyte cell count < 11 × 10^9^ /L	9	1	1	14	5	30 (81%)
Not documented	2			1	1	4 (11%)

^*a*^ All data are presented as the number of patients, if not otherwise specified.

^*b*^ Date of primary surgery not found in medical records.

There were also no differences when comparing the presence of signs of infection at radiology between hip and shoulder PJIs, for example, loosening of the implant or bone resorption, (monomicrobial infections, *P* = 0.23; polymicrobial vs monomicrobial shoulder PJIs, *P* = 0.63) or regarding issues with wound healing disturbances (monomicrobial infections, *P* = 0.06; polymicrobial vs monomicrobial shoulder PJIs, *P* = 0.13,).

In total, 5/13 (38.4%) patients in the hip group and 10/23 (43.5%) patients in the shoulder group had elevated C-reactive protein (CRP) levels, including those with polymicrobial infections. Seven patients in the hip group and nine patients in the shoulder group had CRP levels < 5. A total of five patients (hip, *n* = 1; shoulder, *n* = 4) had no laboratory investigation performed prior to diagnosis and/or treatment (Table 2). The number of patients with elevated CRP did not differ significantly between the hip and shoulder groups (monomicrobial infections, *P* = 1; polymicrobial vs monomicrobial shoulder PJIs, *P* = 0.30) or between patients infected with phylotype IA and those with phylotypes IB/II (*P* = 0.14). Only three patients (one with a hip PJI and two with shoulder PJIs) had elevated blood leukocyte cell count.

### Surgical and antibiotic treatment of patients with PJI caused by *C. acnes*

Among both hip and shoulder patients, the most common surgical procedure was a total revision of the arthroplasty, either a one-step (*n* = 8 and *n* = 9, respectively) or a two-step procedure (*n* = 3 and *n* = 6, respectively) (Table 3).

**TABLE 3:** Treatment and outcome of PJIs in the hip, knee, and shoulder caused by *C. acnes* in 37 patients^*a*^

Treatment and outcome	Hip	Polym. hip	Knee	Shoulder	Polym. shoulder	Total (%)
Surgical intervention						
DAIR	1					1 (3%)
One-stage revision	8		1	8	1	18 (49%)
Two-stage revision	2	1		4	2	9 (24%)
Change of mobile components				1	1	2 (5%)
No revision, only debridement				3	2	5 (14%)
Other^*b*^	1			1		2 (5%)
Outcome						
Implant in place > 1 year after revision, asymptomatic	8	1	1	8	2	20 (54%)
Implant in place > 1 year, still symptomatic				5		5 (14%)
Chronic infection					1	1 (3%)
No implant in place > 1 year after surgery				1		1 (3%)
Loss to follow-up	4			3	3	10 (27%)
Months between revision and last follow-up, median (range)	4 (2–6)			5 (4–5)	5 (4–7)	

^*a*^ All data are presented as number of patients, if not otherwise specified.

^*b*^ Patient primarily had a fracture as indication for surgery, but tissue samples obtained during surgery came back positive for *C. acnes*.

In total, 16 patients received initial intravenous antibiotic treatment and 8 received only oral antibiotic treatment. For eight patients, data regarding antibiotic treatment were not available from the medical records because the treatment was administered from other regions in Sweden. The most common empirical intravenous treatment was beta-lactam antibiotics, sometimes in combination with vancomycin (*n* = 11). Continued intravenous antibiotic treatment involved various antibiotics or combinations such as beta-lactam antibiotics, clindamycin, rifampicin, and/or vancomycin.

Follow-up oral antibiotic treatments for the patients who initially received intravenous treatment were clindamycin (*n* = 2), clindamycin and rifampicin (*n* = 5), amoxicillin (*n* = 1), and various combinations including amoxicillin, rifampicin, fluoroquinolones, clindamycin, and penicillin V (*n* = 5 in total). The medication used for the eight patients who only received oral antibiotic treatment comprised clindamycin (*n* = 2), amoxicillin (*n* = 2), clindamycin + rifampicin (*n* = 2), clindamycin + amoxicillin later changed to amoxicillin only (*n* = 1), and clindamycin + rifampicin later changed to amoxicillin only (*n* = 1).

The median duration of intravenous antibiotic treatment was 8 days (range: 0–85 days), and the median duration of oral antibiotic treatment was 3 months (range: 1–9 months).

Five of the 37 patients did not receive any antibiotic treatment at all, except for routine surgical prophylaxis with cloxacillin. One of them underwent revision surgery because of a fracture in close proximity to the prosthesis, and the remaining four had verified loosening of the implant. Four of the five patients had a one-stage revision, and the remaining patient had the cup revised. According to the patients’ medical records, the suspicion of PJI before revision surgery was low in these cases, and the cultures were reported to be positive for *C. acnes* approximately 2 weeks after revision surgery. Three of the five patients without any antibiotic treatment had growth of *C. acnes* in 2/5 tissue samples, and the remaining two had growth in 4/5 samples. In all five cases, the finding of *C. acnes* was regarded as contamination, and so ,no additional antibiotic treatment was given. One patient in this sub-group had mechanical complications of the prosthesis 10 years after revision and needed a second revision but had no growth of *C. acnes* in tissue samples from the second revision. The remaining four patients were followed for 1–2 years postrevision and had no remaining symptoms and signs noted in their medical records.

### Outcomes among patients with PJIs caused by *C. acnes*

The follow-up period for the clinical evaluation in this study ranged from 3 to 17 years, based on the date for revision surgery. There were no treatment failures in the monomicrobial hip PJI group, but failure was observed in six patients with monomicrobial shoulder PJI (*P* = 0.05) and one additional patient with a polymicrobial shoulder infection. Among patients with shoulder prostheses, five patients had symptoms (predominantly pain and impaired function) >1 year postoperatively. One patient was diagnosed with a chronic infection but still had the implant in place after 1 year, and one patient did not have a functional prosthesis in place. The *C. acnes* isolates associated with failure belonged to SLST classes A (5/7; 71%) and K (2/7; 29%). In comparison, the isolates from patients with successful treatment in the shoulder group belonged to SLST classes A (9/16; 56%), K (3/16; 19%), D (2/16;12.5%), and H (2/16; 12.5%).

Outcome (success vs failure) did not differ significantly between cases with and without a polymicrobial infection (*P* = 0.07), or between patients who received rifampicin at any point during their treatment period (*n* = 8) and those who did not (*n* = 29).

In total, 10 patients were lost to follow-up and were followed for less than 1 year postoperatively. Among these patients, the median time between surgery and the final follow-up was 4 months in the hip group and 5 months in the shoulder group. Seven of the 10 patients had been referred for surgery from neighboring regions. The follow-up of these patients was primarily performed in their home regions, and due to the way the Swedish healthcare system is organized, it was difficult to access their medical data from outside those regions. However, there were no documented symptoms or signs indicating a remaining infection before re-referral, and none of the patients lost to follow-up sought medical care related to their arthroplasties after their final follow-up.

## DISCUSSION

The finding of *C. acnes* in tissue cultures obtained during arthroplasty revision surgery is difficult to interpret, since it may represent a true infection or a contamination. In our previous study from 2019, *C. acnes* isolates obtained from PJIs were compared with *C. acnes* isolates from healthy skin and no statistically significant difference in the prevalence of specific STs among PJI isolates could be identified (34). However, one might question whether all patients in that study had a true PJI, since in several cases, only one isolate was stored per patient and clinical data were not available to further confirm the diagnosis. The present study took a different approach, as we included only *C. acnes* tissue culture-positive cases with multiple isolates of *C. acnes* belonging to the same ST and therefore clearly fulfill the EBJIS criteria for PJI with ≥2 identical microorganisms in multiple samples. We argue that such cases are more likely to be true PJIs caused by *C. acnes*, compared with PJI cases with multiple *C. acnes* isolates belonging to different STs, as one can assume that most PJIs have a single monomicrobial and monotypic infectious trigger.

Seven patients had polymicrobial infections associated with *C. acnes*. In all but one case, the number of positive *C. acnes* tissue samples exceeded the number for the other pathogen, indicating that *C. acnes* was the main pathogen. The clinical data did not differ significantly between the included monomicrobial and polymicrobial infections, and we could not identify any significant differences in outcome.

*C. acnes* isolates from the 37 PJI cases in this study belonged to SLST classes A, C, D, H, and K, which cover the phylotypes IA_1_ (A, C, D), IB (H), and II (K). Most strains belonged to SLST class A, and a few clusters of phylogenetically related class A1 strains were identified, but further analysis showed that all of the included isolates were unrelated. This heterogeneity among the isolates is in concordance with our previous study (34), indicating that there is no specific enrichment of a distinct SLST type, SLST class, or phylotype among PJI-causing *C. acnes*. In other words, many different STs seem to have the potential to cause PJIs. This is to some extent in disagreement with previous studies that have shown a higher prevalence of phylotype IB and II strains among implant-associated infections (21, 35–37).

Even though a phylogenetically diverse set of strains could cause PJI, it is possible that the PJI-causing strains possess unique traits that are important in disease etiology. To evaluate this, we investigated the presence and molecular patterns of specific virulence factors, such as CAMP factors 1 and 2, DsA1, Hyl, and the tad locus-containing plasmid, among our isolates. All isolates contained the genes for CAMP factors 1 and 2, DsA1 and Hyl. The phylogeny of these virulence factors among the *C. acnes* isolates in our study reflected the core genome-based phylogeny. This suggests that PJI isolates do not harbor a specific variant of these virulence factors (e.g., aa specific protein signature) that might be relevant in the context of a PJI.

A previously investigated factor, Hyl, might have an important role in tissue invasion and thus both in the onset of PJI and in inflammatory events. Hyl-IB/II, which is found in phylotypes IB and II, is a variant of hyaluronidase that can catalyze the complete degradation of hyaluronic acid, while the variant found in type IA isolates (Hyl-IA) is only capable of incomplete degradation (26, 27). This has consequences for inflammation, since it has been shown in a murine acne model that strains with the Hyl-IA variant are strongly pro-inflammatory, whereas those with the Hyl-IB/II variants are modestly anti-inflammatory (27). In our study, the Hyl variants were divided according to phylotype, meaning that all PJI-causing phylotype IA strains possessed Hyl-IA and all PJI-causing phylotype IB and II strains possessed Hyl-IB/II. Thus, a specific Hyl variant seems not important in *C. acnes* PJI isolates. Regardless of the ability to partially or totally degrade hyaluronic acid, Hyl could still be an important factor in the pathogenesis of *C. acnes* PJIs.

The tad locus plasmid was identified in only five strains; these isolates belonged to SLST classes A and K, which is in line with previous findings from both PJIs and skin (30, 33, 34). This indicates that the plasmid does not play a major role in *C. acnes* PJI pathogenesis.

The habitat and the low virulence of *C. acnes* and insidious symptoms presented by affected patients are a challenge for the clinician when interpreting a positive culture of *C. acnes* from tissue samples (10, 18, 38). A wide range of symptoms has been described, and many were observed in the patients included in our study, although none of our cases had a sinus tract (39).

Different classifications of PJIs have been proposed over the years. In this study, we used the classification described by Zimmerli and Ochsner in 2003 (11), consisting of early onset (<3 months), delayed onset (3–24 months), and late onset (>24 months) infections, in order to illustrate the diversity of PJIs caused by this low-virulence pathogen. More recently formulated classifications do not distinguish between delayed and late infections and instead classify all infections with onset >4 weeks postoperatively as chronic (13, 40). A study of unspecified PJI of the hip found that >90% of the infections was diagnosed within 30 days (41). The distribution of early infections among our study material was, as expected, very low compared with those results. Six of our patients sought medical attention within 30 days postsurgery (range: 3–29 days), but none was diagnosed with a PJI due to *C. acnes* within 30 days postsurgery. The majority of hip infections in our material were classified as delayed or late. Among shoulder infections, there were a few more patients with early onset infection, but we found no statistically significant difference between shoulder and hip infections. This further strengthens the use of a more diverse system for classification of low-virulence infections.

Approximately half of the patients in both the shoulder and the hip groups did not display elevated inflammatory markers, such as CRP or leucocyte count, indicating that patients with normal inflammatory markers can still have *C. acnes*, PJIs, and polymicrobial PJIs associated with *C. acnes*. This result is important to take into account in the clinical setting, since these markers are frequently relied on for detection of infection (10, 42).

The proportion of patients infected after primary surgery (as opposed to revision surgery) was significantly larger in the shoulder group than in the hip group. *C. acnes* is more prevalent in shoulder PJIs than in other locations, possibly because of its high presence in the sebaceous glands of the torso (15, 43). There have also been findings of *C. acnes* in the dermis and deep tissue of patients undergoing first-time shoulder surgery (18, 44). In addition, *C. acnes* might be able to survive intracellularly, for example, in macrophages, and it is hypothesized that its presence might contribute to low-grade inflammation of the surrounding tissue, both in the shoulder joint and in other organs such as the prostate and spinal discs (18, 30, 45). The abundance of *C. acnes* on the skin of the chest, including the shoulder area, might contribute to the prevalence of *C. acnes* in native shoulder joints and thereby the rate of infection. However, no information is available regarding the presence of *C. acnes* in tissue adjacent to other joints such as hips or knees.

Treatment options for PJIs caused by *C. acnes* or other low-virulence pathogens are a combination of surgical revision and antibiotic treatment, since these infections are chronic when diagnosed. A minimum of 6 weeks of antibiotic treatment is recommended (46). Benzylpenicillin followed by oral amoxicillin is the first line of treatment for PJIs caused by *C. acnes*, according to national guidelines for bone and joint infections (www.infektion.net), which is in line with data from several studies indicating low resistance to beta-lactam antibiotics among *C. acnes* (15, 46, 47). Patients included in the present study were most often empirically prescribed beta-lactam antibiotics, either alone or in combination with vancomycin. The most commonly used oral antibiotic treatments were clindamycin, amoxicillin, and combinations of various antibiotics.

There is no strong clinical evidence of better outcomes from the use of rifampicin in *C. acnes* PJIs (46, 48), even though *in vitro* and *in vivo* studies have shown high susceptibility to rifampicin among *C. acnes* isolates (47) and an additive effect of rifampicin on biofilm eradication when administered in combination with other antibiotics such as daptomycin, vancomycin, and levofloxacin (49). Among the patients included in the present study, there was no statistically significant difference in outcome between those who received rifampicin at any point during their treatment period and those who did not.

In most cases of PJI, the surgical treatment needs to be extensive, involving either DAIR or a one- or two-step exchange procedure. In chronic infections where mature biofilm is present, exchange of prosthetic devices is necessary, and this was the case for most of the patients in the present study. In addition, patients with loosening of components but without preoperative suspicion of infection were revised by one-stage exchange surgery. Successful resolution of *C. acnes* PJIs has previously been achieved in >90% of patients through exchange arthroplasty and long-term antibiotic treatment (50–52). DAIR can be an option in well-selected cases with acute infection, but the risk of treatment failure is higher compared with exchange arthroplasty if the infection is already classified as delayed or chronic (46, 53).

In the present study, no statistically significant difference in outcome was found when comparing patients with monomicrobial hip vs shoulder infections. However, all failures were seen in the shoulder group. There was no statistically significant difference in outcome between patients with mono- and polymicrobial shoulder PJIs, indicating that this diagnostic factor might not be important for outcome. However, clinical data on polymicrobial *C. acnes* PJIs are limited, and there have been some indications that polymicrobial PJIs in general might indicate poorer outcome for the patients (54–56). We identified two severe failures in our study population, including one patient with a chronic infection and one patient with explantation of prosthetic devices, as well as five patients with a prosthesis in place but remaining pain or loss of function of the joint. The rate of poor patient-reported outcomes including pain and decreased range of motion is often high for shoulder infections (51). It is known that the outcome of primary arthroplasty is generally better among patients receiving hip prostheses, compared with shoulder prostheses. Technical factors contributing to postoperative pain and stiffness might include difficulties with humeral component position and alignment, as well as glenohumeral instability or malalignment, possibly leading to erosion of the glenoid (51). Revision surgery because of a PJI is sometimes an even more complicated procedure, with the risk of tissue loss and suboptimal positioning of the components related to the infection. These factors could contribute to the increased risk of poor outcome.

### Limitations and strengths

The selection of patients is both a limitation and a strength of the present study. Cases with possible contamination (i.e., polyclonal findings of *C. acnes*) were excluded, and so, only patients with monoclonal infections were included, that is, ≥2 identical intraoperative tissue samples according to molecular typing. A minimum of five intraoperative tissue samples were collected in most cases, but the number of stored isolates was often lower due to routine procedures. Nevertheless, a mean of four isolates per patient (range: 2–10) was stored and typed. Polymicrobial findings involving suspected co-infections with *S. epidermidis* and *S. saccharolyticus* are not uncommon and may represent both the nature of these bacteria as part of the skin microbiota and the synergistic way they act on the skin and perhaps also in an established infection. Furthermore, since polymicrobial infections are frequent, it can be argued that polytypic infections are also possible; this subject needs further investigation (57, 58).

Our study population was relatively large, with a total of 37 patients, and the follow-up period was generally long. Although some patients were lost to follow-up before the 1-year point, at least 2 years have now passed since their treatment was stopped, and according to their medical records, these patients have not sought any medical attention since their last visit. Even though they do not qualify as successful treatments according to the study definitions, these factors strengthen the probability of success.

### Conclusions

No specific SLST types or identifiable molecular specificity regarding virulence determinants was found among *C. acnes* isolated from PJIs. There were also no differences between isolates from the upper and lower limbs. The treatment of *C. acnes* PJIs is successful in most cases, even when complicated with a polymicrobial infection. However, in comparison to hip infections, shoulder infections showed a higher risk of treatment failure and remaining symptoms 1 year after revision surgery.

## MATERIAL AND METHODS

### Patient selection

Patients (*n* = 122) undergoing revision surgery of a hip, knee, or shoulder arthroplasty during 2005–2019 at the Departments of Orthopedics in Region Örebro County and Region Östergötland, with a PJI where *C. acnes* had been isolated, were included in the present study (*n* = 122).

### Bacterial isolates and culture diagnostics

The culture and species verification of *C. acnes* was performed in accordance with routine diagnostic procedures. Samples were cultured for 5 days in an anaerobic atmosphere (80% N_2_, 10% CO_2_, 10% H_2_) at 36°C on FAA plates [4.6% (wt/vol) LAB 90 Fastidious Anaerobe Agar, LAB M, Heywood, Bury, UK, supplemented with 5% horse blood]. The isolates were characterized by colony morphology, Gram-staining, catalase tests, and indole tests, and selected cases determined to species level by API 20 A (bioMérieux, Marcy-l’Étoile, France) before the implementation of matrix-assisted laser desorption/ionization time-of-flight mass spectrometry (MALDI-TOF MS) with a Microflex LT and Biotyper 3.1 (Bruker Daltonics, Bremen, Germany) in January 2014. All isolates were retrospectively confirmed by MALDI-TOF MS. In addition, the samples were incubated in enrichment broth [2.97% (wt/vol) fastidious anaerobic broth, Lab M, supplemented with 1% (wt/vol) d-glucose] for 7 days. If no growth was detected, the broth was subcultured on a FAA plate for 5 days in an anaerobic environment at 36°C. The isolates were stored in a preservation broth (trypticase soy broth with 0.3% yeast extract and 29% horse serum) at −80°C pending further analysis.

### Single-locus sequence typing

SLST was performed on all isolates from patients with multiple bacterial isolates stored (*n* = 55). DNA was extracted from all isolates, and the SLST fragments were amplified by PCR using 5′PRIME HotMasterMix (5 PRIME, Hamburg, Germany) with 5′-CGCCATCAAGGCACCAACAA- 3′ as forward primer and 5′-ATATCGGCCCGTATTTGGGC-3′ as reverse primer. The thermal cycling started with denaturation at 96°C for 40 sec, and then, 35 cycles were performed at 94°C for 35 sec, 55°C for 40 sec, and 72°C for 40 sec, with a final extension step at 72°C for 7 min. Agarose gel electrophoresis was used to verify the amplified PCR fragments. After PCR purification (NucleoSpin Extract Kit, Macherey-Nagel), the amplicons were Sanger sequenced (GATC Biotech, Cologne, Germany). Forward and reverse sequence reads were aligned, and the quality of the consensus was controlled by manual inspection. STs were assigned to each amplicon using the SLST database (http://medbac.dk), which currently contains 194 STs.

### DNA isolation and whole genome sequencing of *C. acnes*

For genomic DNA extraction from *C. acnes* strains, the MasterPure Gram-Positive DNA Purification Kit (Lucigen) was used as per the manufacturer’s instructions. The concentration and purity of the isolated DNA were first checked with a NanoDrop ND-1000 (Peqlab, Erlangen, Germany); concentrations were determined using the Qubit dsDNA HS Assay Kit as recommended by the manufacturer (Life Technologies GmbH, Darmstadt, Germany). Illumina shotgun libraries were prepared using the Nextera XT DNA Sample Preparation Kit and subsequently sequenced on a MiSeq system using the v3 reagent kit with 600 cycles (Illumina, San Diego, CA, USA) as recommended by the manufacturer. Quality filtering was done with version 0.39 of Trimmomatic (59). Assembly was performed with version 3.14.1 of the SPAdes genome assembler (60). Version 2.2.1 of Qualimap was used to validate the assembly and determine the sequence coverage (61). In total, 37 *C*. *acnes* genomes were sequenced with an average genome coverage of 165-fold; after assembly, the average contig number was 22.4. All genome sequences were deposited in GenBank (see Table S1 for accession numbers).

### Genome sequence data analyses

Gene prediction and annotation of all genomes were performed with PGAP (62). For phylogenomic analyses, the core genome was identified and aligned with the Parsnp program from the Harvest software package (63). All *C. acnes* genomes available from GenBank (*n* = 438 as of March 2023) were used along with the 37 *C*. *acnes* genomes from this study to build a core genome-based phylogeny. Reliable core genome single-nucleotide variants identified by Parsnp were used for the reconstruction of genome-based phylogeny. Phylogenetic trees were visualized using the Interactive Tree Of Life (64).

### Clinical evaluation

To further evaluate the clinical aspects of PJIs caused by *C. acnes*, we examined the patients’ medical records to collect information about age, sex, clinical course, surgical procedure, and antibiotic treatment. All data were generated using a standardized form (see Appendix 1 for details).

### Definitions

Outcome was defined as success or failure. Successful treatment was defined as follows: (i) no signs of infection (laboratory or clinical) 1 year after completing antibiotic treatment, without surgical intervention, and (ii) functioning implant in place 1 year after revision surgery (one step, two step, or DAIR) with no further symptoms (for example, pain, redness, and swelling) or laboratory sign of infection. Failure was defined as follows: (i) having a prosthesis in place after 1 year but still presenting clinical symptoms and/or laboratory findings of infection or (ii) amputation or resection arthroplasty. Loss to follow-up was defined as patients who were followed for less than 1 year after revision surgery, including those who died within 1 year.

### Statistics

Statistical analyses were performed in R studios. In order to evaluate differences between groups, we used Fisher’s exact test or a χ^2^ test for proportions. Statistical significance was defined as *P*  <  0.05. The statistical calculations based on clinical data included only patients undergoing shoulder and hip replacement. No patients undergoing knee replacement were included due to the low number in this group (*n* = 1).

## Data Availability

The data generated and analyzed in the current study are available from the corresponding author upon reasonable request. All genomic data of the Swedish isolates used for this study have been deposited at GenBank with BioProject number PRJNA850913.

## SUPPLEMENTAL MATERIAL

Figure S1 spectrum.00303-24-s0001.tiff Four included patients with multiple sequence types.

Figure S2 spectrum.00303-24-s0002.tiff Ten excluded patients with different STs among their multiple samples.

Figure S3 spectrum.00303-24-s0003.tiff Core genome-based single nucleotide variant analysis.

Supplemental material spectrum.00303-24-s0004.pdf Appendix 1: Form for clinical review of medical records.

Table S1 spectrum.00303-24-s0005.docx All patients, isolates, SLST-results, Genebanknumber, etc.

ASM does not own the copyrights to Supplemental Material that may be linked to, or accessed through, an article. The authors have granted ASM a non-exclusive, world-wide license to publish the Supplemental Material files. Please contact the corresponding author directly for reuse.
