# Supplementary figures and images for: Genomic characterization and clinical evaluation of prosthetic joint infections caused by Cutibacterium acnes
Source: Microbiol Spectr. 2024 Oct 8;12(11):e00303-24. doi: 10.1128/spectrum.00303-24 (PMC11537072; doi:10.1128/spectrum.00303-24)

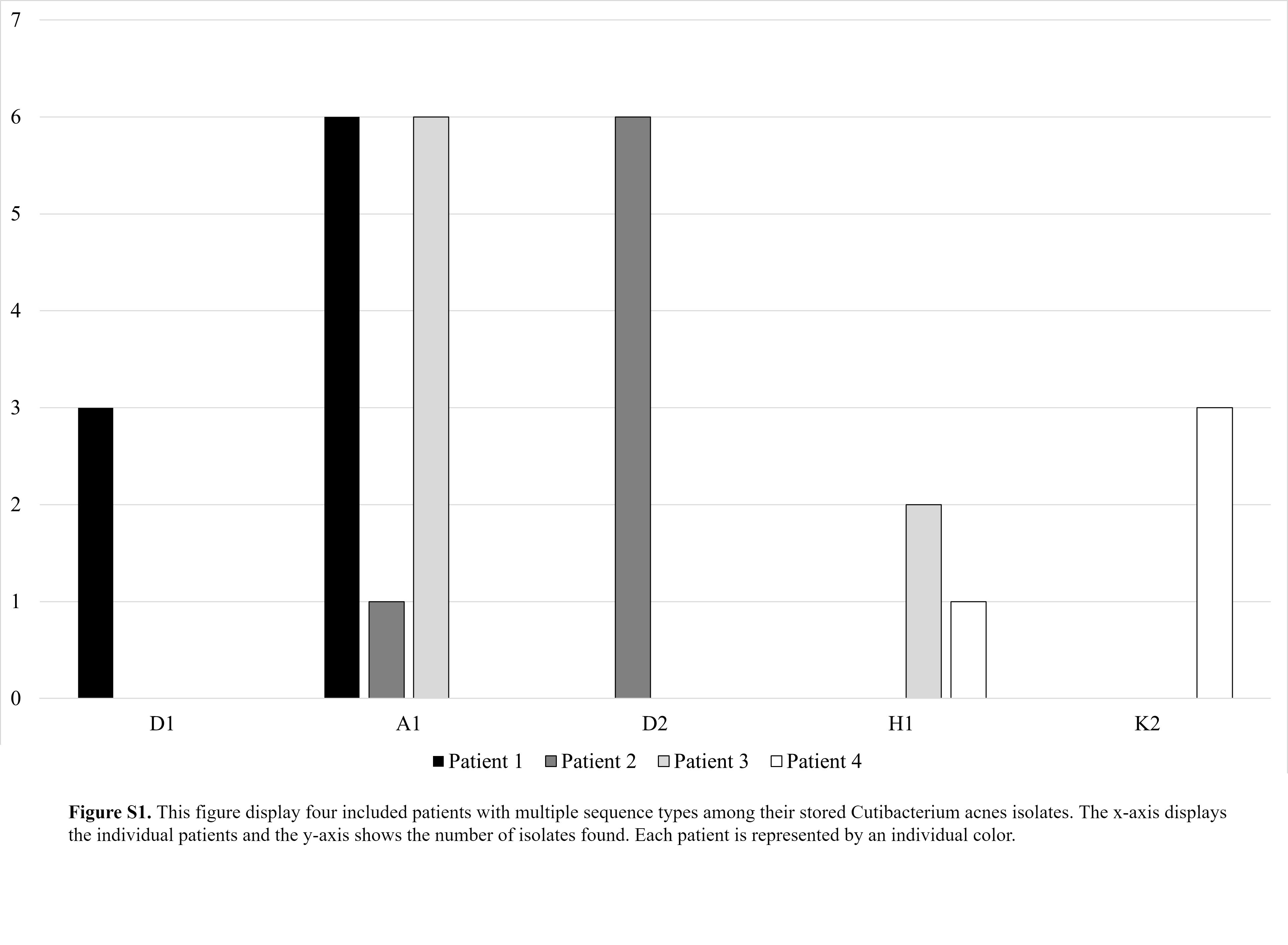

Supplement: Figure S1 — Four included patients with multiple sequence types. [file spectrum.00303-24-s0001.tiff]

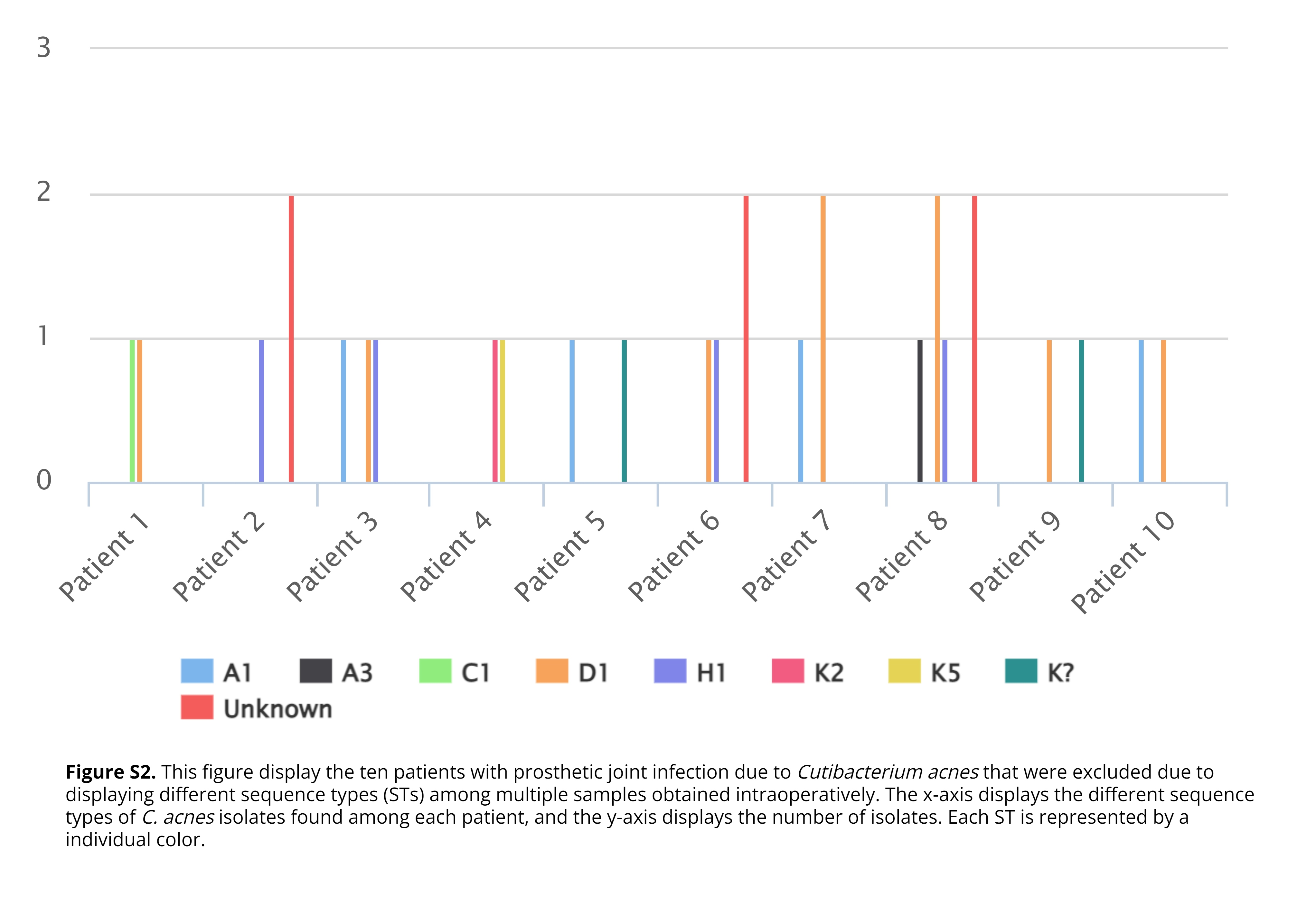

Supplement: Figure S2 — Ten excluded patients with different STs among their multiple samples. [file spectrum.00303-24-s0002.tiff]

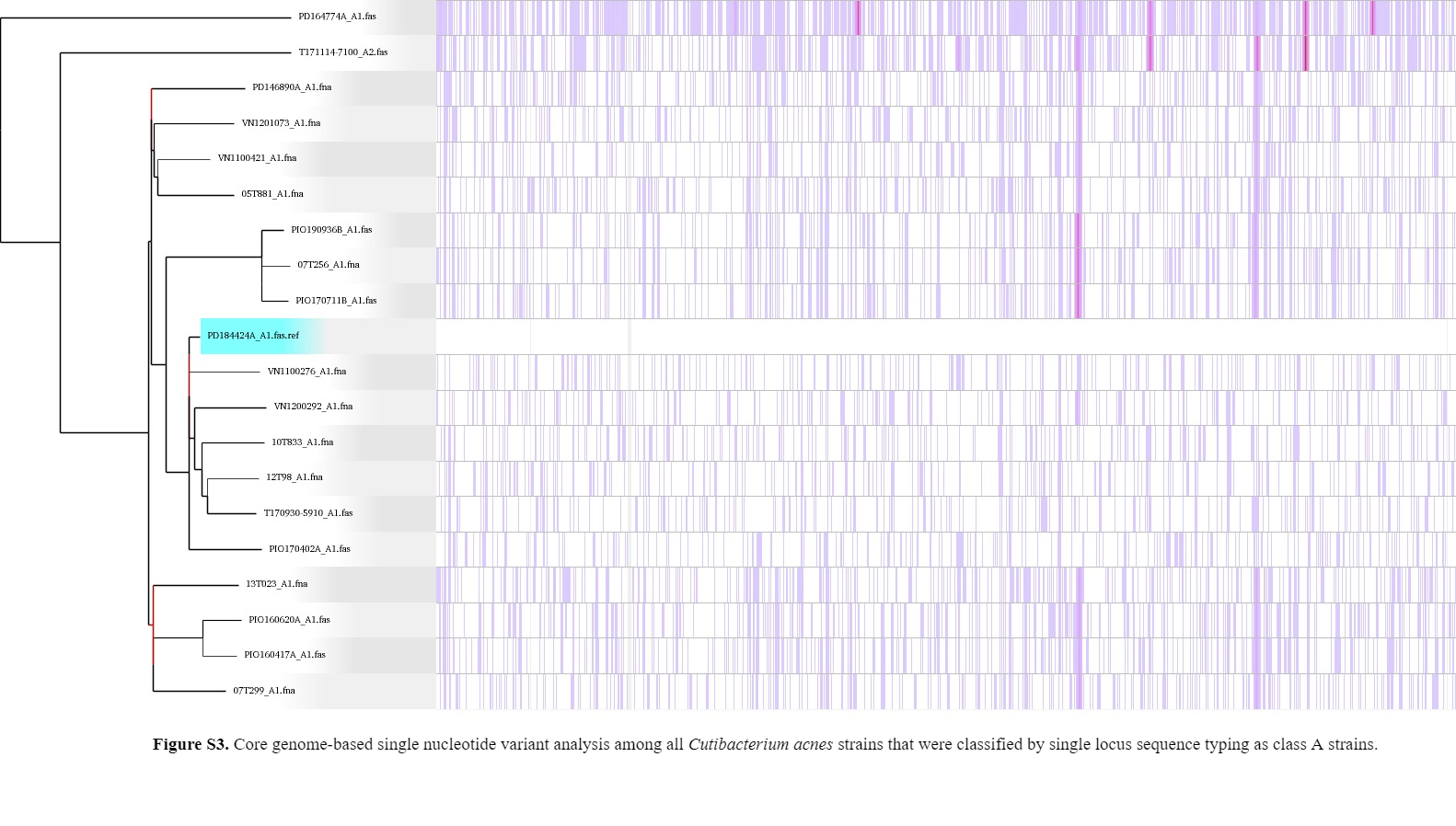

Supplement: Figure S3 — Core genome-based single nucleotide variant analysis. [file spectrum.00303-24-s0003.tiff]
