# Supplementary material for: Genomic characterization and clinical evaluation of prosthetic joint infections caused by Cutibacterium acnes
Source: Microbiol Spectr. 2024 Oct 8;12(11):e00303-24. doi: 10.1128/spectrum.00303-24 (PMC11537072; doi:10.1128/spectrum.00303-24)
Supplement: Supplemental material — Appendix 1: Form for clinical review of medical records. [file spectrum.00303-24-s0004.pdf]

Appendix 1.

Review of medical data – ”C. acnes and prosthetic joint infections”

Patient number: ___________________________ **Lab number**: ___________________________

City: ___________________________________

Contributing disease

☐1. Reumathoid arthritis ☐6. Smoking
☐2. Immunosupression ☐7. BMI >30
☐3. Diabetes ☐8. ASA
☐4. Prior surgery of the joint
☐5. Intraarticular injection
Other: ________________________

Location
☐Left ☐Right
Infected joint
☐Knee ☐Hip ☐Shoulder
Type
☐Cemented ☐Uncementerad ☐Data missing
☐Primary surgery ☐Revision ☐Hybrid

Manufacturer_______________Insicion__________

Diagnostics ☐ x-ray ☐CT ☐ MR ☐Ultrasound
Loosening of prosthetic ☐yes ☐No
Surgical time____ Drainage___
Date primary surgery: ______________________

Duration of symptoms (days): ________________

Date for revision: _______________________

☐. Healing difficulties post surgery

Symptoms as diagnosis

☐1. Secretion ☐
☐2. Pain ☐
☐3. Fever ☐
☐4. Redness ☐
☐5. Fistula ☐

Other: ________________________

Aspiration of synovial fluid
☐Not performed
Joint- white cell count (x 10^9): ________________

Bacterial growth in synovial fluid:__________________________

Cultivation
☐Polymicrobial:________________________

Growth in number of tissue samples/total number of tissue samples: _____/______

CRP (at diagnosis): _______ SR: ________White blood cell count:__

Comments:

Surgical treatment
☐Debriedment, prosthesis remaining
☐Extraction of prosthesis, one stage revision
☐Extrakction of prosthesis, two stage revision with spacer
☐Extraction of prosthesis, two stage revision without spacer
☐Extraction of prosthesis without reimplantation of new prosthesis.
☐Amputation

Prosthesis free intervall in two stage revision
Number of days: _______

Tissue samples collected at revision
Number of samples with bacterial growth/total number of samples: ____/_____
Number of days without antibiotics prior to sugery : _______

Outcome

☐Healed ☐Implant in place ☐Reimplanterad ☐Protesis extracted without reimplantation

☐Chronic infection

☐Relapse ☐Same pathogen ☐Other pathogen

☐Loss to followup, < 12 months follow up post surgery

Date for last follow up: ________________

Antibiotikabehandling

Antibiotika 1 i.v.
☐Isoxazolyl-pc
☐Glykopeptid
☐Klindamycin
☐Daptomycin
☐Aminoglykosid
☐Karbapenem
☐Cefalosporin
☐Trimetoprim-sulfa
☐Tazobactam
☐Bensyl-pc
☐Linezolid
☐Annat
Behandlingstid:____________

Kommentar:

Antibiotika 2 i.v.
☐Isoxazolyl-pc
☐Glykopeptid
☐Klindamycin
☐Daptomycin
☐Aminoglykosid
☐Karbapenem
☐Cefalosporin
☐Trimetoprim-sulfa
☐Tazobactam
☐Bensyl-pc
☐Linezolid
☐Annat
Behandlingstid:____________

Kommentar:

**Antibiotika p.o.**	**1**	**2**	**3**	**4**	**5**	**6**
Rifampicin	☐	☐	☐	☐	☐	☐
Kinoloner	☐	☐	☐	☐	☐	☐
Linezolid	☐	☐	☐	☐	☐	☐
Klindamycin	☐	☐	☐	☐	☐	☐
Fucidinsyra	☐	☐	☐	☐	☐	☐
Trimetoprim-sulfa	☐	☐	☐	☐	☐	☐
Isoxazolyl-pc	☐	☐	☐	☐	☐	☐
Cefalosporin	☐	☐	☐	☐	☐	☐
PcV/Amoxicillin	☐	☐	☐	☐	☐	☐
Annat	☐	☐	☐	☐	☐	☐

Behandlingstid
Start
-
Slut

Orsak till utsättning/biverkningar?

Kommentar:

____________________ ____________________ ____________________
