# Supplementary material for: Genomic characterization and clinical evaluation of prosthetic joint infections caused by Cutibacterium acnes
Source: Microbiol Spectr. 2024 Oct 8;12(11):e00303-24. doi: 10.1128/spectrum.00303-24 (PMC11537072; doi:10.1128/spectrum.00303-24)
Supplement: Table S1 — All patients, isolates, SLST-results, Genebanknumber, etc. [file spectrum.00303-24-s0005.docx]

| **Supplementary table 1.** SLST types, phylotypes, number of positive cultures (including polymicrobial findings) and GenBank accession numbers of the 37 *C. acnes* isolates sequenced in this study. | | | | | | | |
| --- | --- | --- | --- | --- | --- | --- | --- |
| **Patient nr** | **Prosthesis localisation** | **Isolate ID** | **Phylotype** | **ST** | **Accession number in GenBank** | **Positive cultures** | **CRP** |
| 1 | Hip |  |  |  |  | 8/12, *C. acnes* | 26 |
|  |  | VN0900499 | II | K2 |  |  |  |
|  |  | VN0900500 | II | K2 | JANDLB000000000 |  |  |
|  |  | VN0900501 | II | K2 |  |  |  |
|  |  | VN0900502 | II | K2 |  |  |  |
|  |  | VN0900503 | II | K2 |  |  |  |
|  |  | VN0900504 | II | K2 |  |  |  |
|  |  | VN0900505 | II | K2 |  |  |  |
|  |  |  |  |  |  |  |  |
| 2 | Hip |  |  |  |  | 4/5, *C. acnes* | <5 |
|  |  | VN1100276 | IA1 | A1 | JANDLA000000000 |  |  |
|  |  | VN1100278 | IA1 | A1 |  |  |  |
|  |  | VN1100280 | IA1 | A1 |  |  |  |
|  |  |  |  |  |  |  |  |
| 3 | Shoulder |  |  |  |  | 3/3 *C. acnes* | <5 |
|  |  | VN1200291 | IA1 | A1 |  |  |  |
|  |  | VN1200292 | IA1 | A1 | JAVCAK000000000 |  |  |
|  |  | VN1200293 | IA1 | A1 |  |  |  |
|  |  |  |  |  |  |  |  |
| 4 | Knee |  |  |  |  | 5/5 *C. acnes* | <5 |
|  |  | VN1100444 | II | K8 |  |  |  |
|  |  | VN1100446 | II | K8 | JANDKZ000000000 |  |  |
|  |  |  |  |  |  |  |  |
| 5 | Hip |  |  |  |  | 3/7 *C. acnes* | 73 |
|  |  | 05T881 | IA1 | A1 | JANDLR000000000 |  |  |
|  |  | 05T916 | IA1 | A1 |  |  |  |
|  |  |  |  |  |  |  |  |
| 6 | Hip |  |  |  |  | 2/6 *C. acnes* | <5 |
|  |  | 06T1174 | II | K5 | JANDLQ000000000 |  |  |
|  |  | 06T1175 | II | K5 |  |  |  |
|  |  |  |  |  |  |  |  |
| 7 | Hip |  |  |  |  | 2/5 *C. acnes* | <5 |
|  |  | 07T256 | IA1 | A1 | JANDLP000000000 |  |  |
|  |  | 07T259 | IA1 | A1 |  |  |  |
|  |  |  |  |  |  |  |  |
| 8 | Hip |  |  |  |  | 4/5 *C. acnes* | <5 |
|  |  | 07T299 | IA1 | A1 | JANDLO000000000 |  |  |
|  |  | 07T300 | IA1 | A1 |  |  |  |
|  |  | 07T301 | IA1 | A1 |  |  |  |
|  |  | 07T302 | IA1 | A1 |  |  |  |
|  |  |  |  |  |  |  |  |
| 9 | Hip |  |  |  |  | 4/6 *C. acnes* | <5 |
|  |  | 10T833 | IA1 | A1 | JANDLN000000000 |  |  |
|  |  | 10T834 | IA1 | A1 |  |  |  |
|  |  |  |  |  |  |  |  |
| 10 | Hip |  |  |  |  | 9/9 *C. acnes* | 116 |
|  |  | 12T98 | IA1 | A1 | JANDLM000000000 |  |  |
|  |  | 12T99 | IA1 | A1 |  |  |  |
|  |  |  |  |  |  |  |  |
| 11 | Shoulder |  |  |  |  | 4/5 *C. acnes*, 2/5 *S. saccharolyticus* | 113 |
|  |  | 13T023 | IA1 | A1 | JANDLL000000000 |  |  |
|  |  | 13T025 | IA1 | A1 |  |  |  |
|  |  |  |  |  |  |  |  |
| 12 | Shoulder |  |  |  |  | 3/5 *C. acnes*, 3/5 *S. epidermidis* | 14 |
|  |  | PIO130013A | IA1 | D1 | JANDLI000000000 |  |  |
|  |  | PIO130014B | IA1 | D1 |  |  |  |
|  |  |  |  |  |  |  |  |
|  |  |  |  |  |  |  |  |
| 13 | Hip |  |  |  |  | 4/5 *C. acnes*, 3/5 *S. saccharolyticus*, 2/5 *S. capitis* | <5 |
|  |  | PIO140663A | II | K7 | JANDLH000000000 |  |  |
|  |  | PIO140664B | II | K7 |  |  |  |
|  |  | PIO140665B | II | K7 |  |  |  |
|  |  | PIO140666A | II | K7 |  |  |  |
|  |  |  |  |  |  |  |  |
| 14 | Shoulder |  |  |  |  | 3/4 *C. acnes* | <5 |
|  |  | PIO140729A | II | K8 | JANDLG000000000 |  |  |
|  |  | PIO140732A | II | K8 |  |  |  |
|  |  | PIO140731A | II | K8 |  |  |  |
|  |  |  |  |  |  |  |  |
|  |  |  |  |  |  |  |  |
| 15 | Shoulder |  |  |  |  | 4/5 *C. acnes* | Missing data |
|  |  | PIO150050A | II | K8 |  |  |  |
|  |  | PIO150050B | II | K8 | JANDLF000000000 |  |  |
|  |  | PIO150052A | II | K8 |  |  |  |
|  |  | PIO150052B | II | K8 |  |  |  |
|  |  |  |  |  |  |  |  |
| 16 | Hip |  |  |  |  | 5/5 *C. acnes* | Missing data |
|  |  | PIO150252A | IA1 | C1 | JANDLE000000000 |  |  |
|  |  | PIO150253A | IA1 | C1 |  |  |  |
|  |  |  |  |  |  |  |  |
| 17 | Shoulder |  |  |  |  | 9/12 *C. acnes*, 3/12 *S. epidermidis* | Missing data |
|  |  | PIO150263A | IA1 | A1 | JANDLD000000000 |  |  |
|  |  | PIO150262B | IA1 | A1 |  |  |  |
|  |  | PIO150264A | IA1 | A1 |  |  |  |
|  |  | PIO150264B | IA1 | A1 |  |  |  |
|  |  | PIO150265A | IA1 | A1 |  |  |  |
|  |  | PIO150265B | IA1 | A1 |  |  |  |
|  |  | PIO150266A | IA1 | A1 |  |  |  |
|  |  | PIO150267B | IA1 | A1 |  |  |  |
|  |  |  |  |  |  |  |  |
| 18 | Shoulder |  |  |  |  | 9/10 *C. acnes* | 24 |
|  |  | PD146886A | IA1 | A1 |  |  |  |
|  |  | PD146890A | IA1 | A1 | JANDLJ000000000 |  |  |
|  |  | PD146893A | IA1 | A1 |  |  |  |
|  |  | PD146895A | IA1 | A1 |  |  |  |
|  |  |  |  |  |  |  |  |
| 19 | Shoulder |  |  |  |  | 5/5 *C. acnes* | <5 |
|  |  | 13T550 | II | K2 | JANDLK000000000 |  |  |
|  |  | 13T551 | IB | H1 |  |  |  |
|  |  | 13T552 | II | K2 |  |  |  |
|  |  | 13T568 | II | K2 |  |  |  |
|  |  |  |  |  |  |  |  |
| 20 | Shoulder |  |  |  |  | 5/5 *C. acnes* | Missing data |
|  |  | VN1201073 | IA1 | A1 | JANDKY000000000 |  |  |
|  |  | VN1201074 | IA1 | A1 |  |  |  |
|  |  | VN1201075 | IA1 | A1 |  |  |  |
|  |  | VN1201076 | IA1 | New A |  |  |  |
|  |  | VN1201077 | IA1 | New A |  |  |  |
|  |  |  |  |  |  |  |  |
| 21 | Shoulder |  |  |  |  | 8/10 *C. acnes* | 160 |
|  |  | VN1100421 | IA1 | A1 | JAVCAL000000000 |  |  |
|  |  | VN1100422 | IA1 | A1 |  |  |  |
|  |  | VN1100423 | IB | H1 |  |  |  |
|  |  | VN1100424 | IA1 | A1 |  |  |  |
|  |  | AN1100732 | IA1 | A1 |  |  |  |
|  |  | AN1100733 | IB | H1 |  |  |  |
|  |  | AN1100734 | IA1 | A1 |  |  |  |
|  |  | AN1100735 | IA1 | A1 |  |  |  |
|  |  |  |  |  |  |  |  |
| 22 | Shoulder |  |  |  |  | 7/10 C. acnes | 13 |
|  |  | PIO150764A | IA1 | D2 | JANDLC000000000 |  |  |
|  |  | PIO150764B | IA1 | D2 |  |  |  |
|  |  | PIO150765A | IA1 | D2 |  |  |  |
|  |  | PIO150766A | IA1 | D2 |  |  |  |
|  |  | PIO150766B | IA1 | D2 |  |  |  |
|  |  | PIO150767A | IA1 | D2 |  |  |  |
|  |  | PIO150767B | IA1 | A1 |  |  |  |
|  |  |  |  |  |  |  |  |
| 23 | Shoulder |  |  |  |  | 6/9 C. acnes | 62 |
|  |  | T171114-7100 | IA1 | A2 | JANDKP000000000 |  |  |
|  |  | T171114-7102 | IA1 | A2 |  |  |  |
|  |  |  |  |  |  |  |  |
|  |  |  |  |  |  |  |  |
| 24 | Shoulder |  |  |  |  | 5/6 C. acnes | <5 |
|  |  | T170930-5910 | IA1 | A1 | JANDKL000000000 |  |  |
|  |  | T171001-5912 | IA1 | A1 |  |  |  |
|  |  |  |  |  |  |  |  |
| 25 | Shoulder |  |  |  |  | 2/5 C. acnes | <5 |
|  |  | PIO190936B | IA1 | A1 | JANDKO000000000 |  |  |
|  |  | PIO190932A | IA1 | A1 |  |  |  |
|  |  |  |  |  |  |  |  |
| 26 | Hip |  |  |  |  | 4/8 C. acnes | 22 |
|  |  | PIO191018A | IB | H1 | JANDKM000000000 |  |  |
|  |  | PIO191015A | IB | H1 |  |  |  |
|  |  | PIO191014A | IB | H1 |  |  |  |
|  |  | PIO191021A | IB | H1 |  |  |  |
|  |  |  |  |  |  |  |  |
| 27 | Shoulder |  |  |  |  | 6/6 C. acnes | 55 |
|  |  | PIO191191A | IB | H1 | JANDKV000000000 |  |  |
|  |  | PIO191192A | IB | H1 |  |  |  |
|  |  | PIO191193A | IB | H1 |  |  |  |
|  |  | PIO191194A | IB | H1 |  |  |  |
|  |  | PIO191195A | IB | H1 |  |  |  |
|  |  | PIO191196A | IB | H1 |  |  |  |
|  |  |  |  |  |  |  |  |
| 28 | Shoulder |  |  |  |  | 5/5 C. acnes | <5 |
|  |  | PIO160417A | IA1 | A1 | JANDKR000000000 |  |  |
|  |  | PIO160418A | IA1 | A1 |  |  |  |
|  |  | PIO160418B | IA1 | A1 |  |  |  |
|  |  | PIO160419A | IA1 | A1 |  |  |  |
|  |  | PIO160415A | IA1 | A1 |  |  |  |
|  |  |  |  |  |  |  |  |
| 29 | Hip |  |  |  |  | 9/10 C. acnes | 94 |
|  |  | PIO160620A | IA1 | A1 | JANDKS000000000 |  |  |
|  |  | PIO160620B | IA1 | A1 |  |  |  |
|  |  | PIO160621A | IA1 | A1 |  |  |  |
|  |  | PIO160621B | IA1 | A1 |  |  |  |
|  |  | PIO160622B | IA1 | A1 |  |  |  |
|  |  | PIO160623A | IA1 | A1 |  |  |  |
|  |  | PIO160623B | IA1 | A1 |  |  |  |
|  |  | PIO160624A | IA1 | A1 |  |  |  |
|  |  | PIO160624B | IA1 | A1 |  |  |  |
|  |  |  |  |  |  |  |  |
| 30 | Shoulder |  |  |  |  | 7/10 C. acnes, 4/10 S. epidermidis | 5 |
|  |  | PIO161114A | II | New K |  |  |  |
|  |  | PIO161114B | II | New K |  |  |  |
|  |  | PIO161115A | II | New K |  |  |  |
|  |  | PIO161115B | II | New K |  |  |  |
|  |  | PIO161116E | II | New K |  |  |  |
|  |  | PIO161117A | II | New K |  |  |  |
|  |  | PIO161117B | II | New K | JANDKW000000000 |  |  |
|  |  |  |  |  |  |  |  |
| 31 | Shoulder |  |  |  |  | 4/6 C. acnes | Missing data |
|  |  | PIO170402A | IA1 | A1 | JANDKQ000000000 |  |  |
|  |  | PIO170401A | IA1 | A1 |  |  |  |
|  |  | PIO170404A | IA1 | A1 |  |  |  |
|  |  | PIO170400A | Unknown | Unknown |  |  |  |
|  |  |  |  |  |  |  |  |
|  |  |  |  |  |  |  |  |
|  |  |  |  |  |  |  |  |
| 32 | Shoulder |  |  |  |  | 4/5 C. acnes | 5 |
|  |  | PIO170632A | II | New K | JAVCAJ000000000 |  |  |
|  |  | PIO170631A | II | New K |  |  |  |
|  |  | PIO170634A | II | New K |  |  |  |
|  |  | PIO170635A | II | New K |  |  |  |
|  |  |  |  |  |  |  |  |
| 33 | Shoulder |  |  |  |  | 3/5 C. acnes, 2/5 S. epidermidis | 20 |
|  |  | PIO170711B | IA1 | A1 | JANDKT000000000 |  |  |
|  |  | PIO170710A | IA1 | A1 |  |  |  |
|  |  | PIO170709C | IA1 | A1 |  |  |  |
|  |  |  |  |  |  |  |  |
| 34 | Shoulder |  |  |  |  | 5/5 C. acnes | <5 |
|  |  | PIO180773A | IB | H1 |  |  |  |
|  |  | PIO180774A | IB | H1 |  |  |  |
|  |  | PIO180775A | Unknown | Unknown |  |  |  |
|  |  | PIO180776A | Unknown | Unknown |  |  |  |
|  |  | PIO180777A | IB | H1 | JANDKU000000000 |  |  |
|  |  |  |  |  |  |  |  |
| 35 | Hip |  |  |  |  | 5/5 C. acnes | <5 |
|  |  | PIO180960A | IA1 | D1 |  |  |  |
|  |  | PIO180961A | IA1 | D1 | JANDKN000000000 |  |  |
|  |  | PIO180962A | IA1 | D1 |  |  |  |
|  |  | PIO180959A | IA1 | D1 |  |  |  |
|  |  |  |  |  |  |  |  |
|  |  |  |  |  |  |  |  |
| 36 | Shoulder |  |  |  |  | 10/10 C. acnes, 2/10 S. epidermidis | 60 |
|  |  | PD164774A | IA1 | A1 | JANDKK000000000 |  |  |
|  |  | PD164773A | IA1 | D1 |  |  |  |
|  |  | PD164775A | IA1 | A1 |  |  |  |
|  |  | PD164780A | IA1 | D1 |  |  |  |
|  |  | PD164777A | IA1 | D1 |  |  |  |
|  |  | PD164776A | IA1 | A1 |  |  |  |
|  |  | PD164779A | IA1 | A1 |  |  |  |
|  |  | PD164772A | IA1 | A1 |  |  |  |
|  |  | PD164778A | IA1 | A1 |  |  |  |
|  |  |  |  |  |  |  |  |
| 37 | Shoulder |  |  |  |  | 4/4 C. acnes | 92 |
|  |  | PD184424A | IA1 | A1 | JANDKX000000000 |  |  |
|  |  | PD184425A | IA1 | A1 |  |  |  |
